# Global Management of Anal Fissure: Results from the ISUCRS 2022 Snapshot Audit

**DOI:** 10.3390/jcm15124677

**Published:** 2026-06-16

**Authors:** Audrius Dulskas, Joseph Nunoo-Mensah, Richard Fortunato, Majid Huneidy, Dursun Bugra, Varut Lohsiriwat, Tomas Aukstikalnis, Narimantas E. Samalavicius

**Affiliations:** 1 Institute of Clinical Medicine, Faculty of Medicine, Vilnius University, LT-03101 Vilnius, Lithuaniatomas.aukstikalnis@mf.vu.lt (T.A.); narimantas.samalavicius@gmail.com (N.E.S.); 2 National Cancer Institute, LT-08406 Vilnius, Lithuania; 3 Department of Colorectal Surgery, King’s College Hospital Foundation NHS Trust, London SE5 9RS, UK; joseph.nunoo-mensah@nhs.net; 4 Cleveland Clinic, London SW1X 7HY, UK; 5 Department of Colorectal Surgery, Allegheny General Hospital, Pittsburgh, PA 15212, USA; rfortunato03@gmail.com; 6 Department of General Surgery, The Vehbi Koç Foundation (VKV), American Hospital, 34365 Istanbul, Turkey; dursunbugra@yahoo.com; 7 Division of Colon and Rectal Surgery, Department of Surgery, Faculty of Medicine Siriraj Hospital, Mahidol University, Bangkok 10700, Thailand; bolloon@hotmail.com; 8 Department of Surgery, Vilnius Republic University Hospital, LT-04130 Vilnius, Lithuania

**Keywords:** anal fissure, chronic anal fissure, fissure treatment, lateral internal sphincterotomy

## Abstract

**Background**: Anal fissure is a common benign condition, yet its management varies widely. The International Society of University Colon and Rectal Surgeons (ISUCRS) conducted a global snapshot audit to describe contemporary real-world management patterns. **Methods**: During a 2-week period (June–July 2022), 56 colorectal surgeons from 21 countries prospectively recorded data for consecutive patients presenting with anal fissure. Exclusion criteria included inflammatory bowel disease, pregnancy or lactation, psychiatric disorders, immunosuppression, and anorectal sepsis. Acute fissure was defined as symptoms <6 weeks without sentinel pile; chronic fissure as >6 weeks or fibrotic edges/sentinel pile. The “Cure” was defined as complete symptom resolution or healed fissure on clinical or tele-follow-up. **Results**: A total of 302 patients were analyzed (mean age 41 ± 13 years; 52% women). Acute fissure was present in 42%, chronic in 58%. Conservative treatment (dietary advice, stool-softeners, topical agents, botulin toxin, pelvic-floor training) was initiated in 236 (78%) patients, while 66 (22%) underwent surgery, most commonly lateral internal sphincterotomy (LIS). At 8-week follow-up, 73% of patients treated conservatively and 88% of those treated surgically achieved clinical resolution of symptoms or healed fissure. **Conclusions**: Global management of anal fissure remains heterogeneous. Most surgeons favor conservative measures such as first-line therapy, reserving LIS for chronic or refractory fissures. Standardized definitions and outcome reporting are needed to improve comparability and guide future international guidelines.

## 1. Introduction

Anal fissure is one of the most common causes of anal pain and bleeding, with 235,000 new cases occurring every year in the United States alone [1]. It accounts for an overall incidence of 1.1 per 1000 person-years and an average lifetime risk of 7.8% [2].

Anal fissure is described as a longitudinal tear in the anoderm of the anal canal distal to the dentate line. It is believed that this pathology occurs due to an overdistention or disease of the anal mucosa, which leads to a laceration of the anoderm [3]. It is believed that the posterior commissure has a poor blood supply, therefore, predisposing it to ischemia [4,5,6].

Anal fissures are usually managed conservatively [7], whether it is done with the use of calcium channel blockers (Nifedipine or Diltiazem), Nitrates, warm sitz bath, oral painkillers, or Botulin Toxin. All of which have been deemed acceptable with few adverse effects and high cure rates. Guidelines set by the American Society of Colon and Rectal Surgeons supported the use of the previously mentioned conservative treatment methods and urged medical professionals to use non-surgical treatment modalities as a first-line treatment since they are considered safe and have few side effects [8].

Surgically, however, lateral internal sphincterotomy (LIS) remains the gold standard to treat anal fissures permanently, more often in chronic cases [9], with a low rate of complications and a reduced cost burden. Whether it is performed using an open or closed approach, both can achieve similar recovery and complication rates [10].

Although anal fissure is a common condition, there is a relative paucity of high-quality data describing real-world management patterns across different healthcare settings. Most available evidence derives from randomized trials, systematic reviews, and guideline recommendations that focus on the efficacy of individual interventions rather than actual clinical practice. Previous studies have demonstrated the effectiveness of conservative therapies and lateral internal sphincterotomy in controlled settings, often reporting high healing rates [8,11,12]. However, population-based and survey data suggest that treatment approaches vary substantially in routine practice, including differences in the use of botulin toxin, topical therapies, and surgical timing [13]. These studies are frequently limited by single-center design, regional scope, or retrospective methodology, and, therefore, do not adequately capture global variation in management. Moreover, the absence of international guidelines increases variation in management, which may affect treatment outcomes, access to care, and recurrence rates.

Our international snapshot audit, coordinated by ISUCRS, sought to provide an overview of contemporary management of anal fissure worldwide, identify regional differences, and evaluate outcomes of conservative and surgical strategies.

## 2. Materials and Methods

This study was approved by the Vilnius Regional Biomedical Research Ethics Committee (Lithuania). Additional approvals from participating institutional ethics boards were obtained where required. After all approvals were issued according to each nation’s laws and consent to participate in the study was obtained by signature from patients, a prospective snapshot audit was performed. 

All the members of ISUCRS who saw at least five patients with anal fissures per month were invited to participate in the study. The inclusion period was any two weeks between June and July 2022. All the consecutive patients were included. Patient data from 21 countries were obtained through the cooperation of 56 doctors and compiled into an electronic database. 

All the participants, before the patient enrollment, underwent online training on anal fissure diagnosis and classification. All the consecutive patients coming to the clinic for anal pain/bleeding, diagnosed with fissure using digital rectal examination and/or endoscopic evaluation (subjective classification), were included in the study. Moreover, the state of fissure (acute vs. chronic) was decided by the treating physician upon examination (the presence of sentinel piles or thick borders was a sign of chronic fissure). Acute fissure: symptoms <6 weeks without sentinel pile. Chronic fissure: symptoms > 6 weeks or with fibrotic edges/sentinel pile. Cure: complete symptom resolution or healed fissure on examination or tele-follow-up. LIS: lateral internal sphincterotomy. Exclusion criteria: inflammatory bowel disease, active perianal sepsis or fistula, pregnancy or lactation, psychiatric disease impairing consent, immunosuppression, and tuberculosis.

The demographic information was collected from the medical records of the patients, and the American Society of Anesthesiologists’ (ASA) classification system assessed the comorbidities associated with the anal fissure. All records of the past medical history, obstetrics history, vaginal delivery, number of vaginal deliveries, history of episiotomy, and history of perineal tear were taken into consideration.

To assess the effect of the intervention on the anal fissures, the patient’s current symptoms were recorded, and a pain visual analog scale was used to assess the severity of the pain. Higher VAS scores indicate greater pain intensity. Patients diagnosed at the time of the current intervention were considered the acute case, and those who had an anal fissure for more than six weeks (or the fissure had sentinel piles or harder borders upon examination) were regarded as the chronic case.

Wexner score was used to assess the continence [14]. The number 0 referred to patients who had no incontinence, and the number 20 was given to patients who had the worst continence.

### 2.1. Treatment and Follow-Up

Initial management was conservative unless the fissure was chronic or recurrent after prior medical therapy. Conservative therapy included dietary advice, fiber or stool-softeners, topical nitrates or calcium-channel blockers, and botulin toxin injection when indicated.

Surgery was considered for refractory cases and consisted mainly of LIS, with fissurectomy or advancement flap in selected patients. Follow-up was performed by the treating surgeon in person or via secure tele-consultation 8 weeks after inclusion.

All the details of the patient’s demographic data, pre-and post-treatment details, and outcomes were noted. The success of the current treatment that was given to patients was assessed at an 8-week follow-up (after the treatment initiation).

### 2.2. Statistical Analysis

This report has been prepared in accordance with the guidelines set by the STROBE (Strengthening the reporting of observational studies in epidemiology) statement for observational studies. Student’s t-test was used for normal, continuous data, Mann–Whitney U test for non-normal continuous data or Chi-squared test for categorical data. Data analysis was undertaken using R Studio V3.1.1 (R Foundation, Boston, MA, USA).

## 3. Results

### 3.1. Patient Demographics

**Figure 1:**
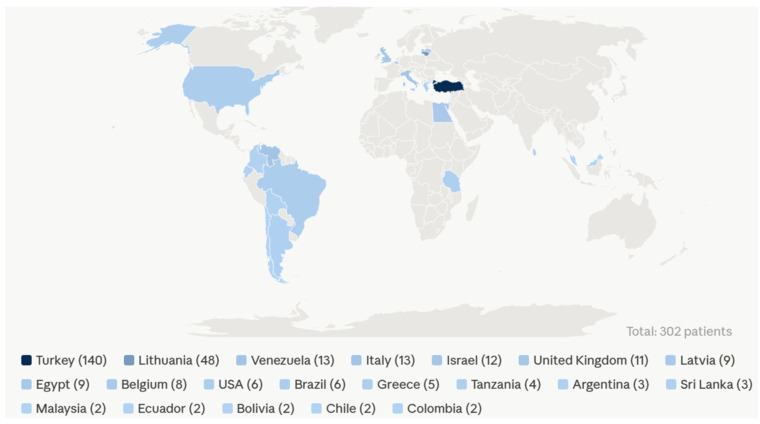
Geographical distribution of included patients.

Of 302 patients analyzed (geographical distribution can be seen in Figure 1), the mean age was 41 ± 13 years (range 18–76); 52% were female, indicating about equal sex distribution. Acute fissures accounted for 42%, chronic fissures for 58%. The difference between men and women was not statistically significant (*p* = 0.47). ASA I was assessed in 198 patients (65.6%), ASA II was found in 93 individuals (30.4%), while ASA III and ASA IV were recorded in nine and two (0.7%) subjects, respectively. Of the 302 included patients, 268 (88.7%) had available follow-up data at 8 weeks, while 34 (11.3%) were lost to follow-up.

Based on the medical history relevant to our study, several patients were grouped according to the similar pathologies they experienced (Table 1).

Focusing on the female cohort, 95 females were found to have had obstetrics history before this visit with 78 patients having an account of vaginal deliveries, out of which 1 (1.2%) of patient having zero vaginal deliveries, 25 (32%) of individuals with one delivery, 35 (46%) with two deliveries and 11 (14%) with three vaginal deliveries. Six females (7%) had four or more vaginal deliveries. Moreover, 48 female patients were found to have had a history of episiotomy, while two patients had perineal tears (the grade could not be specified).

### 3.2. Clinical Presentation

**Figure 2:**
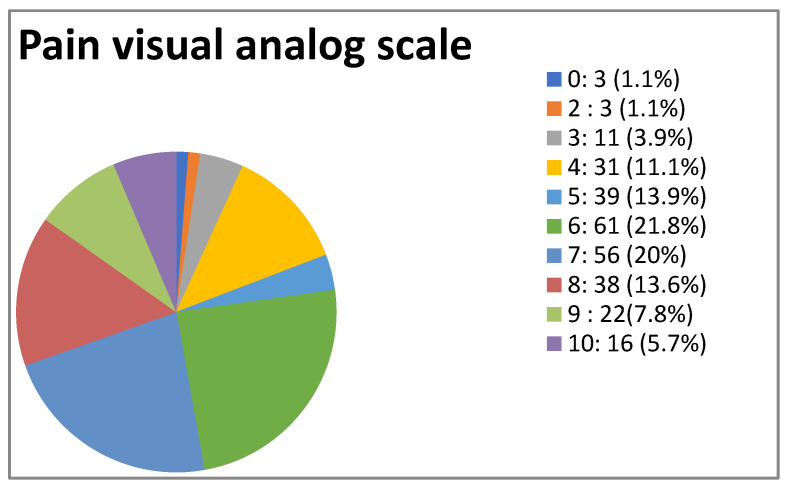
Pain visual analog scale for patients with anal fissure.

Leading symptoms were painful defecation (*n* = 280, 92.7%), followed by anal bleeding (*n* =194, 64.2%) and painful bleeding during defecation (*n* = 182, 60.2%). The severity of the pain experienced by patients at the time of inspection was taken into account and categorized using the pain visual analog scale (Figure 2). Almost all the patients experienced some degree of pain (98.9%); bleeding occurred in 64%, and pruritus in 22%. Information on stool consistency was available for 190 (63%) patients: constipation in 58%, normal in 38%, diarrhea in 4%.

### 3.3. Previous Treatments

**Figure 3:**
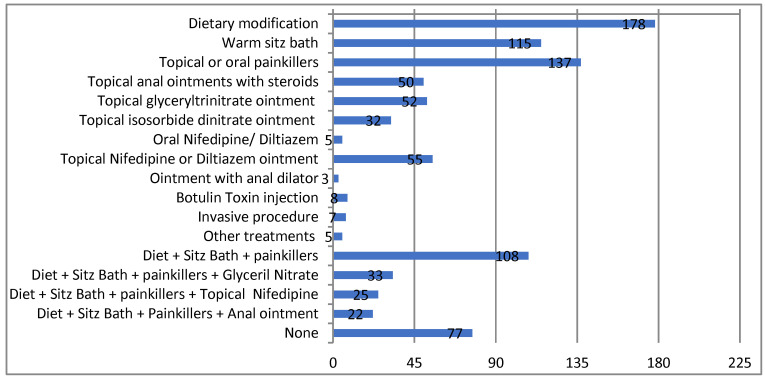
Conservative treatments for patients with anal fissure prior to his visit.

**Figure 4:**
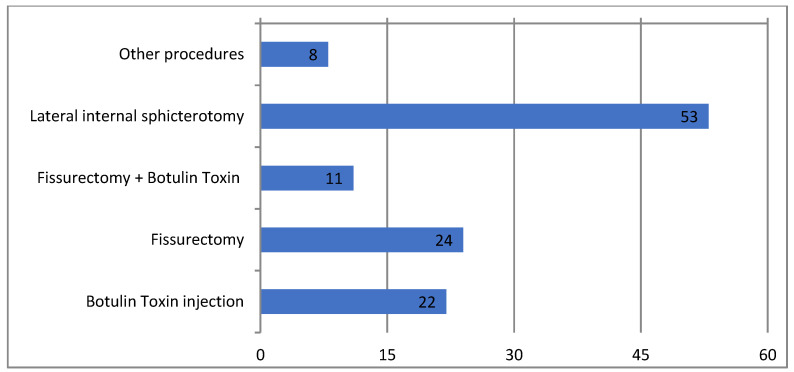
Surgical treatments for patients with anal fissure before the visit.

Before this, several patients underwent various treatment methods, ranging from conservative (Figure 3) to surgical approaches (Figure 4) (the majority of them had a persistent disease). Moreover, 142 of them (the recurrent cases) had some surgical treatment, with 53 of them having LIS. Moreover, five patients had fissurectomy and later LIS. Thirty-four patients had more than one surgical procedure.

### 3.4. Current Management

**Figure 5:**
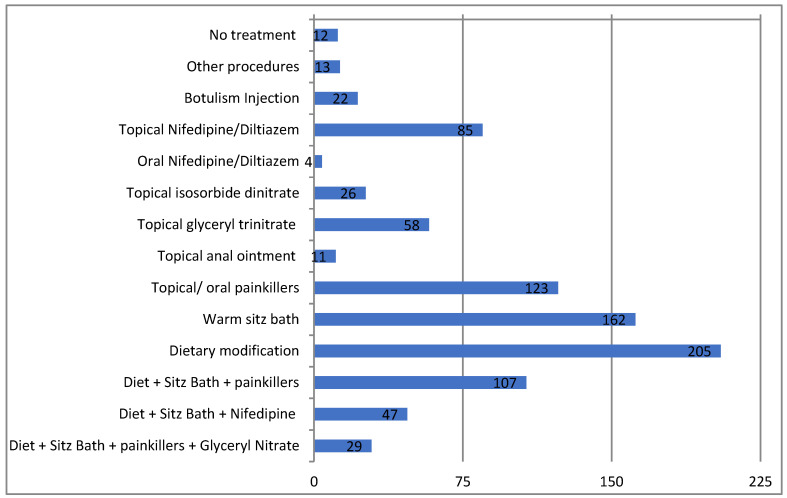
Conservative treatment used during the current visit for patients with anal fissure.

Conservative management was chosen in 236 (78%) patients. Topical nifedipine or diltiazem was the most common (61%), followed by nitrates (24%), botulin toxin (7%), and pelvic-floor training (8%) (Figure 5). Italy stands out with 62% receiving office Botox—far higher than anyone else. Latvia sends 100% of its current patients to surgery—the only country to do so. Venezuela relies almost entirely on conservative measures: dietary mod (92%), sitz baths (77%), and ISDN (77%), with zero surgery. Lithuania uses topical CCB in 70% of current encounters—unique in the cohort. USA reports 50% “none”—possibly reflecting referral patterns where patients are being assessed rather than actively treated.

### 3.5. Surgical Details

Surgical treatment was undertaken in 66 (22%) patients, mainly LIS (*n* = 55, 83%), fissurectomy (*n* = 8), and advancement flap (*n* = 3). In surgical intervention, which totaled 256 interventions (some patients underwent multiple interventions (previous + current procedures), 155 (60.5%) patients were given the ambulatory surgical procedure, whereas 101 (39.5%) patients were given inpatient admission with several anesthetic techniques used during interventions (Table 2).

Sixty-six patients underwent different surgical procedures, ranging from anal dilatation (22) to botulin toxin injection (24) and others. Latvia performs fissurectomy in 89% of surgical cases—almost exclusively. Belgium opts for fissurectomy + Botox in 88%—a combined approach rarely used elsewhere. Brazil combines fissurectomy (67%) and LIS (83%)—the highest LIS rate in the cohort. LIS is the dominant procedure in Lithuania (23%), Turkey (21%), and Egypt (33%), while being essentially absent in Latvia and Belgium. Anal stretch appears notably in Greece (40%) and Brazil (17%), but is absent in most countries—reflecting the declining use of this technique globally.

### 3.6. Complications

Eighteen patients experienced treatment-related complications. The most common adverse event was headache associated with topical nitroglycerin use (*n* = 15, 83%). Other documented events included allergic reaction to nifedipine (*n* = 1), hypotension after topical diltiazem (*n* = 1), perineal sepsis (*n* = 1), postoperative bleeding (*n* = 2), urinary retention (*n* = 1), anesthetic-related complications (*n* = 1) (latter four complications were surgery related), and other minor events (*n* = 4).

### 3.7. Follow Up

Upon assessing the Wexner fecal incontinence score during our study, we noticed a change in the pretreatment vs. post-treatment score. In fact, the average Wexner score for individuals in the range of 0 to 10 in the pretreatment was 1.07 ± 3.1 compared to a post-treatment average of 1.31 ± 1.25. However, regarding patients being scored in the range of 11 and above, only the pretreatment cohort indicated an average of 11.61, with no individual post-treatment possessing a score higher than 10. Of the 71 paired patients, 62 improved (85.9%), 7 were unchanged (9.9%), and only 3 worsened (4.2%). Of the total patients, 268 received follow-ups within eight weeks after the current treatment, of which 224 (84%) patients had the actual visit with the doctor, and 44 (16%) had the virtual visit. After the treatment, 119 (44.4%) of patients were free of anal fissures. Eighty-six (32.2%) showed marked improvements with no medications after intervention; 46 (17.1%) showed gradual improvement, needing medication now and then. Sixteen (6.0%) of patients reported no change in the condition. One had worse (0.3%) symptoms. None of the patients required extended hospitalization or rehospitalization.

## 4. Discussion

This international snapshot audit provides real-world insight into the management of anal fissure across diverse healthcare systems. The key finding of this study is the substantial global variation in treatment strategies, despite the availability of well-established clinical guidelines.

First, we observed that conservative treatment remains the predominant initial approach, used in 78% of patients. This is broadly consistent with guideline recommendations, which advocate stepwise management starting with non-surgical therapies. However, the type and combination of conservative treatments varied considerably, including differences in the use of topical agents, botulin toxin, and pelvic floor interventions. This variability likely reflects differences in local expertise, resource availability, healthcare system structure, and physician preference rather than evidence-based standardization. In a study by Tavakoli-Dastjerdi et al., constipation was considered a major cause of fissures [15], and it was, therefore, recommended that avoiding certain foods and commercial baked goods may be beneficial. In contrast, our study noted that the benefits of dietary changes were correlated with stool consistency, not with any specific food intake or avoidance. It is important to clearly specify that dietary advice should be aimed at achieving soft stools. This is the only condition that can promote fissure healing, in combination with the application of various ointments. Therefore, it is not only important to provide advice, but also to ensure the result. That is, the stools should not be too soft or too hard; otherwise, healing with conservative treatment may be impossible.

The second most used conservative treatment identified in this study was the use of a warm sitz bath, which has been speculated to have an analgesic effect as well as improve healing by relieving sphincter spasm. A study undertaken by Jensen et al. [16], who used warm sitz baths along with unprocessed bran to treat anal fissures in 96 patients, argued that this treatment combination yielded the same results as topical analgesics and anti-inflammatory ointments while avoiding their side-effects and costs [16].

Another study performed by Alnasser et al. [17] on 519 patients has shown that the use of a conservative management protocol consisting of a salty warm sitz bath three times daily, 2 g glycerin suppositories per rectum 20 min before defecation, and bulk-forming fiber daily yielded complete fissure healing in 379 (70.3%) patients with a duration range from 3–7 weeks. The remaining 160 (29.7%) patients who did not heal ultimately had surgical intervention with a 0% recurrence rate. 

Topical or oral painkillers were also a conservative method of treatment chosen by a large group of our physicians. The use of topical or oral calcium channel blockers has shown effectiveness in treating anal fissures by alleviating painful symptoms and by vasodilation, therefore, accelerating the healing process. This has led others to stress that the use of topical painkillers should be considered before considering surgical options [18].

The major limitations of painkillers are cost, temporary benefits, and higher relapse rates, leading some to recommend LIS for patients who have failed to respond to first-line conservative therapy, or for those who relapse with medical management [19]. A prospective controlled trial that compared the effect of 2% diltiazem and LIS for treatment of chronic anal fissures found LIS was more effective for complete healing at 6 weeks (96% vs. 71%), and for pain relief [18].

The use of botulin toxin was not frequently used by our physicians, with 6.6% using it to treat chronic anal fissures and only 0.7% in acute cases. When used as first-line therapy for chronic anal fissures, botulin toxin produces comparable results to topical therapies; however, when used as second-line therapy after topical therapies, the use of botulin toxin would only slightly improve healing rates [7]. A pooled analysis of studies indicated a 13.5% increase in the absolute rate of healing and a 38% increase in the rate of healing in comparison to placebo or Lidocaine alone [18].

A survey from the American Society of Colon and Rectal Surgeons (ASCRS) [12] showed that most surgeons (90%) were using 50-100U of botulin toxin, with most respondents (64%) injecting the internal sphincter and a majority of participants (53%) injecting it into 4 quadrants of the anal canal circumference. Most procedures were performed under Monitored Anesthesia Care (56%). In fact, an increase in the usage of botulin toxin by our physicians was noticed when treating chronic cases, with 6.6% in comparison with only 0.7% used in acute cases. A similar study supported the use of botulin toxin in chronic, uncomplicated anal fissures with an increased sphincter tone due to its tolerability, ability to be administered in outpatient settings, and low probability of causing incontinence [20].

Second, surgical management was performed in 22% of patients, predominantly using lateral internal sphincterotomy (LIS). While this aligns with current recommendations for chronic or refractory fissures, the relatively high proportion of surgical interventions—particularly in a short observational window—may indicate variation in thresholds for surgery across regions. This suggests that, although guidelines are widely known, their implementation may not be uniform, and decision-making may be influenced by local practice patterns and patient factors. A study by Altomare et al. [13] stated that for acute anal fissures, conservative treatment can provide a cure in 87% of cases, but only 50% in cases of chronic anal fissures. They concluded that if conservative treatments fail to provide a definitive treatment, physicians usually resort to the use of invasive procedures. This is also supported by recently issued guidelines [7] and was seen in our survey. A LIS was the procedure of choice for physicians participating in this study [21] due to its effectiveness and low risk of fecal incontinence. This was found to be in line with other studies regarding healing, patient satisfaction, and low recurrence rates [22,23]. Our study consists of many patients undergoing any kind of surgical intervention. This could be explained by the fact that many patients with recurrent or persistent diseases were included in our survey.

Fecal incontinence after LIS ranges from ‘sometimes’ to ‘frequently’ and includes lack of control of flatus (35.1%), soiling of underclothing (22%), and accidental bowel movements (5.3%)—but following sphincterotomy, these numbers might be overestimated [24].

According to our survey, patients who had treatment did not develop worsening fecal incontinence. Changes in the Wexner score before and after treatment showed improvement from 96% of patients who had a score of 10 or lower (indicating continence) to 100% with a score of 10 or lower, showing the efficacy and safety overall of fissure treatments. However, short follow-up, lack of instrumental testing, and telemedicine as a follow up mean should be taken into account not to underestimate the incidence.

A main issue expressed by researchers who have undertaken similar studies on anal fissures was the length of the follow-up period, where one study had 6-week follow-up while another had a 4-week follow-up; this has unfortunately proven to be insufficient. At least an 8-week post-treatment follow-up visit is required for the assessment of the status of the fissure. Most patients show improvement in this period, as shown in our study. The period of 8 weeks is essential to conclude which intervention has a better response and can be used for future treatment [25].

Although this global survey included patients with anal fissure from all continents, it has some limitations. First, patient selection may be influenced by surgeon participation, and there was no external monitoring of case submission. Second, adherence to prescribed conservative treatment (including stool regulation and topical therapy) could not be objectively verified. Third, follow-up was limited to 8 weeks, preventing assessment of long-term recurrence or continence outcomes. Fourth, sphincter tone was not routinely evaluated using manometry, limiting objective comparison of functional outcomes. Finally, some follow-up was performed via telemedicine without examination, meaning healing assessment in those cases was symptom-based rather than clinical—this could overestimate the healing rates. Due to heterogeneity and design constraints, we avoided multivariable modeling. Approximately 11% of patients were lost to follow-up, which may introduce attrition bias. It is possible that patients with persistent symptoms or complications were less likely to attend follow-up, potentially leading to an overestimation of treatment success.

This study’s strengths are its global scope and the use of prospective, real-time data collection (snapshot audit).

## 5. Conclusions

Worldwide, most patients with anal fissure continue to be managed conservatively, with surgical treatment reserved for chronic or refractory cases. Despite regional practice variation, short-term outcomes appear favorable. Standardized definitions and reporting frameworks are needed to support future comparisons and guideline development.

## Figures and Tables

**Table 1:** The past medical history of patients included in the study.

Past Medical History	Number of Patients
Hemorrhoids	82
Vaginal childbirth	54
Anal Fistula	10
Anorectal trauma	5
HIV/AIDS	3
Psoriasis at genital area	1
Genital Herpes	1
Other	15
None	164

**Table 2:** Anesthetic technique used during surgical interventions (*n* = 256).

Anesthetic Technique	Number of Patients Who Underwent the Mentioned Technique
Intravenous anesthesia	23 (8.9%)
Local anesthesia	33 (12.8%)
A combination of local and intravenous anesthesia	36 (14%)
Regional block	75 (29.3%)
General anesthesia	89 (34.7%)

## Data Availability

The data presented in this study are available on request from the corresponding author.

## Supplementary Materials

The following supporting information can be downloaded at: https://www.mdpi.com/article/10.3390/jcm15124677/s1, 2022 International Society of Universities of Colon and Rectal Surgeons (ISUCRS) Collaborating Group.
