# Supplementary material for: Global Management of Anal Fissure: Results from the ISUCRS 2022 Snapshot Audit
Source: J Clin Med. 2026 Jun 16;15(12):4677. doi: 10.3390/jcm15124677 (PMC13302191; doi:10.3390/jcm15124677)
Supplement: Supplementary file 1 [file jcm-15-04677-s001.zip › jcm-4320262-supplementary.pdf]

2022 International Society of Universities of Colon and Rectal Surgeons (ISUCRS) Collaborating Group

Darija Soldatenkova

Ainārs Boks

Georgijs Kočiašvili

Adnan Özpek

Ahmad Fardi Sulaiman

Alisina Bulut

Alon Pikarsky

Alper Sozutek

Argyrios Ioannidis

Arnold Leiboff

Barış Gülcü

Can Saraçoğlu

Cemal ULUSOY

Cengizhan Yigitler

Cristopher Varela

Dakshitha Wickramasinghe

Enver Kunduz

Edmund Leung

Ergin Erginöz

Francisco Quinteros

Giuseppe Brisinda

Hakan Bolukbasi

Haktan Ovul Bozkir

Yasir Musa Kesgin

Julio Picón Ponce

Kağan Zengin

Korhan Tuncer

Mehmet Faik Özcelik

Mehmet Gulmez

Mehmet Selçuk Gerger

Michail Klimovskij

Mirza Faraz Saeed

Muhammed Tahir Akca

Muratcan FIRAT

Naciye Cigdem Arslan

Nail Omarov

Nikas Samuolis

Paola De Nardi

Paras Barta

Sander Van Hoof

Sefu Juma Uledi

Semra Demirli Atici

Serhan Yilmaz

Serhat Meriç

Servet Sezgin Uludag

Sezai Leventoglu

Sofia Xenaki

Tamer El Zalabany

Valdemir José Alegre Salles

Vita Klimasauskiene

Xavier Delgadillo

Zurabs Kecbaja
